# Innovations, Challenges, and Regulatory Pathways in Cultured Meat for a Sustainable Future

**DOI:** 10.3390/foods14183183

**Published:** 2025-09-12

**Authors:** Imad Khan, Jiage Sun, Wanmei Liang, Rui Li, Kit-Leong Cheong, Zehua Qiu, Qiuyu Xia

**Affiliations:** 1 Guangdong Provincial Key Laboratory of Aquatic Product Processing and Safety, Guangdong Province Engineering Laboratory for Marine Biological Products, Guangdong Provincial Engineering Technology Research Center of Seafood, Guangdong Provincial Engineering Technology Research Center of Prefabricated Seafood Processing and Quality Control, College of Food Science and Technology, Guangdong Ocean University, Zhanjiang 524088, China; imadk5577@gmail.com (I.K.); jiagesun@163.com (J.S.); kakabula@163.com (W.L.); liruihn@163.com (R.L.); klcheong@gdou.edu.cn (K.-L.C.); 19967319679@163.com (Z.Q.); 2 Collaborative Innovation Center of Seafood Deep Processing, Dalian Polytechnic University, Dalian 116034, China

**Keywords:** cultured meat, cellular agriculture, stem cells, tissue engineering, bioreactors, scaffolds

## Abstract

Cultured meat is produced through cellular agriculture and tissue engineering and has emerged as a promising alternative to conventional animal-based meat production. Cultured meat, produced through cellular agriculture and tissue engineering, offers a sustainable alternative to conventional meat production. This review outlines the potential of diverse stem cell sources, including satellite cells, embryonic stem cells, and induced pluripotent stem cells, for producing muscle and adipose tissue. Advances in bioprocess development, biomaterials, and bioreactor design are discussed, with an emphasis on scalability, cost reduction, and regulatory considerations. Despite progress, key challenges remain: replicating the nutritional composition and sensory qualities of conventional meat, developing serum-free media, and ensuring consistent large-scale production. Recent studies report cost reductions of up to 90% in culture media and successful bioreactor expansions beyond 50 L, yet industrial translation is still limited. Consumer acceptance and clear regulatory frameworks are also critical for commercialization. Future work should focus on integrating cellular innovations with scalable technologies to overcome current bottlenecks and accelerate market readiness.

## 1. Introduction

The growing global population, projected to reach 10 billion by 2050 [1], will drive an increasing demand for food [2]. As a result, it is crucial to develop sustainable, affordable, and practical solutions. Global meat consumption is projected to rise by around 1.4% annually [3], highlighting the need for alternatives such as cellular agriculture, which enables the production of animal-derived food products in vitro through tissue engineering [1,3]. Tissue engineering, initially designed to restore the function of injured organs and tissues [4], has also become a valuable in vitro tool for disease modeling and drug development [5]. More than 15 years ago, this technology was proposed for food applications, leading to the first cultured meat prototype in 2013 [6].

Cultured meat aims to address the challenges of industrial livestock farming by mitigating environmental and ethical concerns [7]. The Intergovernmental Panel on Climate Change highlights the urgent need to reduce conventional animal product consumption to minimize climate risks. Yet, consumer resistance to dietary change remains strong [8]. Cultured meat, derived from stem cells that generate skeletal muscle and fat tissue, provides a viable alternative by reducing reliance on livestock. Like other emerging technologies, it offers benefits in three key areas: sustainability, animal welfare, and public health.

**Figure 1:**
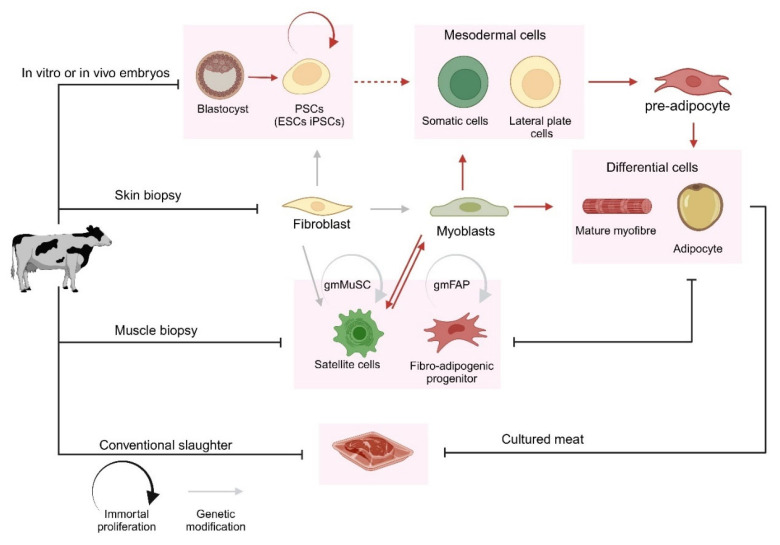
Sources of cells and their differentiation pathways required for the production of cultured meat. The process of deriving adipogenic and myogenic cells from pluripotent stem cells (PSCs), whether sourced from induced pluripotent stem cells (iPSCs) or embryonic stem cells (ESCs), requires additional steps (illustrated by dashed arrows) compared to their differentiation from fibro-adipogenic progenitor cells or progenitor satellite cells. Black arrows represent manual cell handling, whereas red arrows indicate reagent-induced in vitro differentiation or in vivo development. Grey arrows represent genetic modifications, with grey circular arrows specifically highlighting modifications used for the immortalization of cell populations.

Animal-derived meat consists mainly of skeletal muscle (≈90%), fat and connective tissues (≈10%), and a small fraction of blood vessels [9]. Over time, skeletal muscle and fat tissues have been engineered for applications such as transplantation for muscle loss and muscular dystrophy [10], soft tissue repair [11], and drug screening [12]. Skeletal muscle tissue primarily contains myofibers, which are protein-rich structures forming the fundamental component of both muscle and meat. It also includes adipocytes, which influence tenderness and flavor, and fibroblasts, which produce extracellular matrix (ECM) to enhance texture [13]. Because adipocytes and myofibers are post-mitotic, cell expansion depends on progenitor or stem cells. While early efforts relied on fibroblasts, recent advances focus on stem cells, which are more practical and scalable due to their capacity to generate the essential cellular components of meat. The sources of these cells and their differentiation pathways are illustrated in Figure 1.

The production of cultured meat still faces major challenges, particularly the need for scalable and cost-effective methods using edible, non-animal-based materials. Production costs remain high, with the culture medium—supplemented with expensive growth factors—representing the greatest expense. Alternative approaches, including serum proteins, plant-based growth factors, and recombinant technologies, are being explored, but remain in early development [14]. Replicating the nutritional composition and sensory attributes of conventional meat, including texture and thickness, is also a significant challenge [15].

This review provides a comprehensive analysis of the technological, regulatory, and societal aspects of cultured meat production. It examines recent progress in cell selection, culture media optimization, and tissue engineering, alongside the role of biomaterials and bioreactors in scaling production. The article further addresses regulatory challenges and consumer acceptance issues, while highlighting current bottlenecks and future research directions necessary to establish cultured meat as a sustainable and practical alternative within global food systems.

## 2. The Concept of Cultured Meat and How It Transforms Food

Cultured meat aims to reproduce the properties of conventional meat by utilizing tissue engineering and stem cell cultivation techniques. The concept was first proposed in 19th-century utopian literature and took practical form in 2013 with the world’s first laboratory-grown meat prototype [16]. Since then, terminology has evolved, with expressions such as clean meat, lab-grown meat, cell-based meat, and cultured meat often used interchangeably. For clarity, this review primarily uses “cultured meat,” while “cell-based meat” is applied in U.S. regulatory contexts where it appears in legal texts.

**Figure 2:**
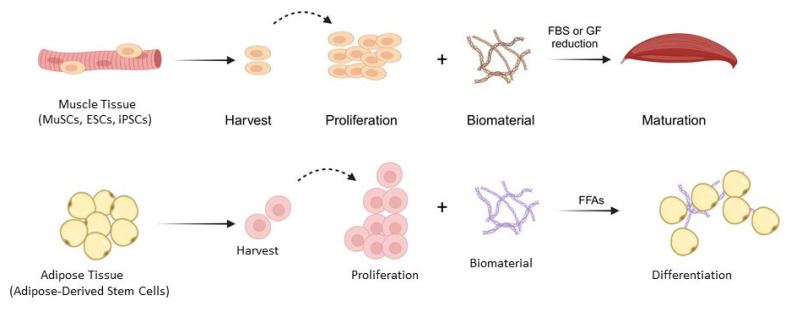
The idea of cultured meat. Stem cells are extracted from mature muscle tissue and adipose tissue precursors before being expanded. Using a specialized differentiation protocol and a gel-based biomaterial, these cells develop into adipose tissue structures and mature muscle fibers. Muscle maturation is supported by a serum-free differentiation medium containing growth factors (GFs) at one-tenth of their initial concentration or by progressively reducing fetal bovine serum (FBS) levels from 20% to 2%. Meanwhile, stem cells derived from adipose tissue differentiate when exposed to free fatty acids (FFAs). Solid arrows indicate the sequential process, whereas dashed arrows indicate proliferative expansion of cells.

Cultured meat production belongs to the expanding field of cellular agriculture, which employs cell-based technologies to create animal-derived alternatives such as seafood, milk, and leather. Advances in stem cell biology have enabled cells to be isolated from living animals through biopsy, expanded in vitro, and directed to differentiate into muscle or fat cells [6]. In most tissue-engineering approaches, biomaterial scaffolds provide the three-dimensional structure and support necessary for developing tissues with sensory and nutritional properties similar to conventional meat. Strategies for replicating meat range from synthesizing specific muscle proteins to constructing complex muscle structures containing fibers, adipose tissue, connective tissue, and, in some cases, vascular or neural components arranged in patterns that closely resemble natural meat (Figure 2). Building on this conceptual foundation, the following sections examine the technical innovations in stem cell biology, culture media optimization, biomaterials, and bioprocessing that are driving the future of cultured meat.

## 3. Selection of Appropriate Cell Type for Producing Cultured Meat

Various cell types can serve as sources for cultured meat production, as summarized in Table 1. Skeletal myocytes are the primary cell type, complemented by smaller populations of fibroblasts, endothelial cells, and adipocytes. The chosen initial cell type must be capable of differentiating into the diverse cell types present in mature meat and exhibit a high proliferation potential essential for large-scale production. Pluripotent cells, such as induced pluripotent stem cells (iPSCs) and embryonic stem cells (ESCs), are particularly suitable for this purpose, as they can proliferate indefinitely and differentiate into nearly any cell type [17]. Additional key sources include adult stem cells (ASCs) such as fibro-adipogenic progenitor cells (FAPs) [18], muscle satellite cells (MuSCs) [19], and mesenchymal stem cells (MSCs) [20]. These cells have the potential to differentiate into specific types of cells depending on their origin of tissue. Although ASCs offer advantages such as rapid and efficient differentiation with minimal external influence and their proliferation potential remains relatively limited [21].

### 3.1. Pluripotent Stem Cells (PSCs)

PSCs, including ESCs and iPSCs, have the capacity for self-renewal and can differentiate into nearly any cell type. Their unique features, such as unlimited self-renewal and a rapid propagation rate, are particularly advantageous for meeting the substantial doubling time requirements of cultured meat. This capability is crucial for establishing cell banks and eliminating the need for repeated biopsies. Cell banks are highly characterized for growth consistency, sterility, and reproducibility to ensure a reliable and safe manufacturing process. Additionally, PSCs are cost-effective, since a single vial of thawed cells can generate multiple production batches. Furthermore, in bioreactors, PSCs can achieve higher cell densities than satellite cells and MSCs in 2D culture due to their smaller size, which enables easier scale-up before cell seeding. PSCs can be cultured in suspension to form cell aggregates or grown on edible microcarriers for enabling 3D culture in bioreactors [23]. These cells can maintain consistent growth throughout their production period by supporting numerous population doublings. To date, their ability for indefinite proliferation raises concerns regarding potential genetic and epigenetic alterations, which highlight the need for rigorous monitoring of genetic stability throughout production [24].

Moreover, PSCs can differentiate into all three germ layers, thereby enabling the production of diverse cell types essential for meat cultivation, including fibroblasts, myocytes, endothelial cells, and adipocytes. They can also give rise to other important cell types, such as hepatocytes and osteoblasts. Their versatility eliminates the need to produce and maintain multiple separate cell lines for various meat components. PSCs are vital as a source of myocyte precursors, collagen-producing cells, and adipocytes, all of which contribute to the texture and sensory characteristics of cultured meat. Therefore, optimizing PSCs in small bioreactors is important. However, efficient cell differentiation protocols for these cell types, particularly in non-human animal species, are still under development.

Furthermore, ESCs can originate from the inner mass of a blastocyst, a structure that forms 8–9 days after fertilization in bovines. Ethically, no animal needs to be killed or even harmed in this process compared to other sources of cells. However, the derived ESC is difficult to maintain because these cells can spontaneously differentiate, which requires the addition of specific growth factors to the culture [25]. ESCs have been derived from species that can already be used for cultured meat production including chickens, fish, and bovines. For instance, chicken ESCs are used by Super Meat to produce cultivated chicken meat, while Aleph Farms uses bovine ESCs to develop cultivated beef, which was grown in suspension in an animal component-free medium. These ESCs were developed into collagen-producing cells and muscle for a thin-cut beef steak production [26].

#### PSC Genetic Modification

One strategy to simplify the complex multi-stage differentiation protocols for PSCs is to genetically modify them to enhance adipogenic and myogenic gene expression. This method has been demonstrated by activating the MYOD gene in human-induced pluripotent stem cells (hiPSCs) using a transposon-based system, successfully inducing myotube formation [27]. Lentiviral transduction effectively facilitated the myotube formation in human iPSCs (hiPSCs) and porcine iPSCs (piPSCs) by inducing MYOD expression, supplemented with fetal bovine serum (FBS) or specific growth factors [28]. Lentiviral-mediated Paired Box 7 (PAX7) overexpression enhanced differentiation into satellite cell-like structures and supported myotube formation in hiPSCs and human embryonic stem cells (hESCs) [29]. Moreover, cells with genetic and structural characteristics resembling mature white adipocytes were made through lentiviral-induced peroxisome proliferator-activated receptor γ (PPAR-γ) expression in mesenchymal progenitors [30]. Additionally, the use of inducible vectors such as doxycycline offers improved control over the differentiation process, which is a significant advantage of direct differentiation through genetic modification. This approach lays the foundation for the development of safer methods for future food production [31].

Genetic modification is an effective method for stimulating stem-cell differentiation, but it also raises concerns about the potential for undesired mutations. iPSCs are produced by expressing transcription factors such as SOX2, OCT4, C-MYC, and KLF4, or by reprogramming somatic cells (e.g., white blood cells or skin fibroblasts) using various techniques including episomes, viral vectors, proteins, or mRNA [32]. These iPSCs have been successfully developed for cultured meat production from species such as pigs [33], chickens [34], fish [35], and bovines [36]. Companies like Meatable and HigherSteaks are using bovine and porcine iPSCs to produce meat products. Meatable uses its proprietary OPTi-OX technology to differentiate iPSCs, derived from reprogrammed hematopoietic stem cells (HSCs) into muscle and fat cells [37].

While ESCs and iPSCs share similar abilities to proliferate and differentiate, iPSCs retain epigenetic memory [37]. This characteristic can be beneficial when the source cells are closely related to the differentiated cells of interest, but it may pose challenges when the target differentiated cell differs from the source cell. Additionally, reprogramming can lead to phenotypic modifications, and the reprogramming process is not always fully efficient [38]. All reprogramming methods except for transient transfection methods (such as proteins or mRNA), produce genetically modified iPSCs due to genome editing, meaning each will require some form of labeling. Unlike ESC derivation, genetic modifications are not obligatory.

### 3.2. ASCs

ASCs are undifferentiated progenitor cells located in specific tissues and organs of adult animals. These multipotent cells have the ability to differentiate into a limited range of cell types based on their tissue of origin. Their limiting proliferative potential of 50–60 divisions (limited by the age of the animal) follows the Hayflick-limit [39]. Consequently, multiple biopsies are often necessary to maintain the production of commercial cultured meat. Ensuring safety, consistency, and reproducibility requires repeated characterization tests, which in turn introduce variability into the process. ASCs have multiple types, which are elaborated below.

#### 3.2.1. Muscle Stem Cells and Satellite Cells

The main component of skeletal muscle is myofibers, which account for approximately 90% of its mass [40]. In conventional meat, these fibers store myofibrillar proteins that provide essential amino acids, iron, minerals, vitamins (A, E, and B), fatty acids, and glycogen reserves [41]. While myofibers generated from stem cells may structurally resemble natural muscle, their ability to accumulate these nutritional components depends largely on the culture environment and may not fully replicate the composition of traditional meat [42].

**Figure 3:**
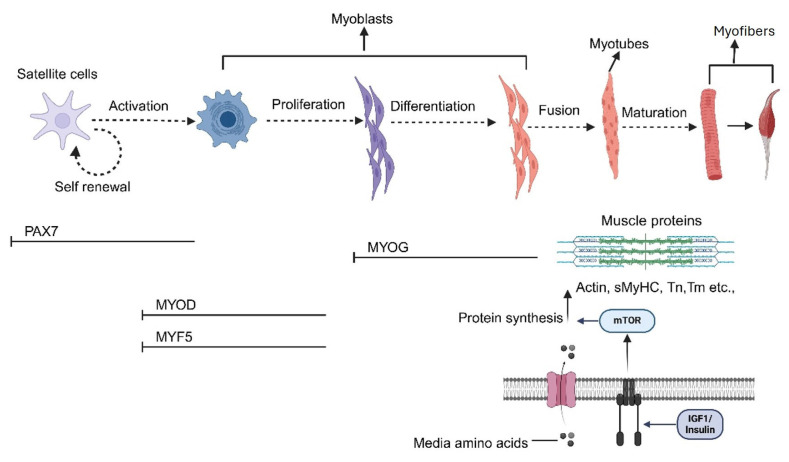
An overview of myocyte differentiation starts with MuSCs and progresses through maturation and myogenesis, leading to protein synthesis. Muscle protein synthesis is regulated by insulin or insulin-like growth-factor 1 (IGF1) through the mTOR signaling pathway, using amino acids from the culture medium. As myofibers mature, contractile proteins such as troponin (Tn), skeletal muscle myosin heavy chain (sMyHC), actin, and tropomyosin (Tm) are produced. In the later stages of differentiation, MYOG plays a crucial role in the fusion of myoblasts into myotubes and their terminal differentiation. MYF5 and MYOD act as transcription factors to initiate proliferation and myogenesis, while PAX7 serves as the primary marker for satellite cells, which are decreasing upon activation. Solid arrows indicate the sequential progression of myogenesis, while dashed arrows indicate alternative or regulatory processes such as self-renewal and stage-to-stage transitions.

MuSCs or satellite cells are a form of ASCs situated on the outer edges of muscle fibers that play a crucial role in producing new myonuclei in postnatal muscle. The quiescent state of MuSCs is associated with the expression of the transcription factor PAX7 (Figure 3). The differentiation, proliferation, and eventual fusion of activated myoblasts are regulated by myogenic regulatory factors. In addition, established protocols for isolating and culturing MuSCs in humans and mice can be adapted for species used in cultured meat production, such as bovine, porcine, and chicken. Moreover, cryopreservation dishes are important for MuSC functionality preservation and cell bank formation [43]. The process of seeding and isolating MuSCs from mature muscle for cultured meat production involves inherent variations. Furthermore, within the same species, MuSCs may differ in their ability to expand and differentiate. For instance, Belgian Blue and Limousin cattle have been reported to maintain their differentiation ability for much longer than other breeds [44]. Additionally, out of nine porcine muscles, the psoas major and extensor carpi radialis yield the highest number of MuSCs, though their availability differs among muscle types [45]. Their expansion potential is also affected by the additional in vivo cell cycles undergone by the donor animal [46]. In mammals, male muscle mass is typically larger than female, and testosterone has been implicated in regulating MuSC numbers in boars [47]. Due to these variations, further research is needed to determine the best animal sources for an abundant supply of MuSCs.

#### 3.2.2. MSCs

MSCs are commonly extracted from adipose tissue or bone marrow, though they can also be sourced from other tissues, such as skeletal muscle [48]. There are several potential sources for the production of cultured meat; for example, MSCs from bovines, chickens, pigs, and fish have been isolated successfully for the formation of cultured meat. These cells mainly aim to develop into osteoblasts, adipocytes, and chondrocytes, but also have limited potential to fuse into skeletal muscle [49]. Consequently, MSCs are an important resource both for the formation of adipocytes and skeletal muscle for cultured meat applications.

#### 3.2.3. Adipogenic Precursors and Adipogenic Stem Cells

Meat quality is largely determined by its fat content and muscle proteins [50]. Although fat can be stored in muscle cells, adipocytes are primarily responsible for producing intramuscular fat, which constitutes approximately 80% of the total fat in meat [1]. Sensory attributes such as taste, texture, juiciness, and color are strongly linked to intramuscular fat content [51]. For cultured meat products to compete in the market, they must include palatability features that replicate the fat characteristics of traditional meat. Unlike plant-based protein alternatives, cultured meat has the potential to replicate the fat profiles of animal meat by using animal stem cells, rather than simply mimicking animal fats [52]. Additionally, incorporating exogenous fatty acids like oleic acid into the culture media could enhance both the health benefits and flavor of cultivated fat. Moreover, the essential polyunsaturated fatty acids (PUFAs) derived from the phospholipid bilayers of cultivated fat could play a crucial role in meeting the body’s PUFA requirements, which are essential for maintaining immune system function and brain health.

Mature adipocytes can be produced from MSCs, fibro-adipogenic progenitor cells (FAPs), and adipose-derived stem cells (ADSCs) [53]. ADSCs and FAPs have similar stem-cell potential to MSCs, but they are found in different anatomical locations. MSCs are present in the bone marrow, FAPs are sourced from the perimysium, and ADSCs originate from adipose tissue. MSCs are among the most thoroughly researched stem cell types primarily due to their capacity for adipogenesis [54]. MSCs are usually obtained by culturing bone marrow in dishes or flasks to expand the adherent cell population. In contrast, FAPs and ADSCs are extracted from muscle or fat tissue through enzymatic digestion, typically using type II collagenase, followed by centrifugation. This process produces a cell pellet containing the stromal vascular fraction, which includes immune cells, pericytes, and preadipocytes such as ADSCs and FAPs. To increase purity, preadipocytes can be expanded or isolated by fluorescence-activated cell sorting (FACS) using adipogenic precursor markers like CD117+, Lin+, CD31+, Sca+, CD34−, PDGF-α+, and CD29+ [54].

**Figure 4:**
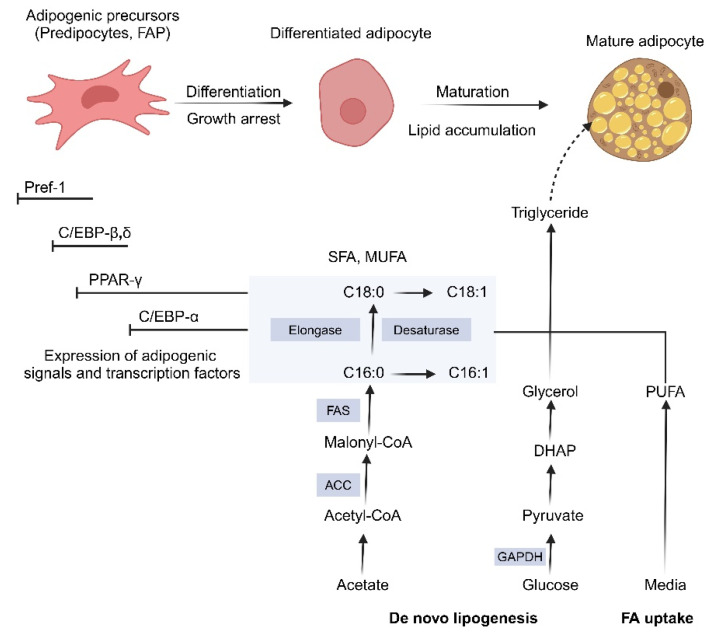
A brief summary of maturation (lipogenesis) and adipogenic differentiation (adipogenesis), starting from adipogenic precursor cells. Through fatty acid (FA) absorption and de novo lipogenesis, monounsaturated fatty acid (MUFA), saturated fatty acid (SFA), and polyunsaturated fatty acids (PUFAs) accumulate. This process is determined by the activation of lipogenic and glycolytic genes, including acetyl-coenzyme A carboxylase (ACC), glyceraldehyde-3-phosphate dehydrogenase (GAPDH), fatty acid elongase, fatty acid synthase (FAS), fatty acid-binding protein (FABP), and Δ9 desaturase, which are regulated by transcription factors such as C/EBP-α and PPAR-γ. During early differentiation, adipogenic signals like C/EBP-δ and C/EBP-β increase, while the expression of preadipocyte factor 1 (Pref-1) declines. Solid arrows indicate active metabolic or differentiation steps, whereas the dashed arrow indicates lipid (triglyceride) accumulation as an outcome of maturation.

Evaluation of preadipocyte adipogenic potential can be determined by evaluating the expression profile of transcription factors and the cell cycle. Transcription Factor Zfp423 is critical for the initial differentiation into preadipocytes during the transition from the multipotent state of MSCs, FAPs, and ADSCs [55]. In bovine muscle-derived FAPs, Zfp423 is identified as a key marker of highly adipogenic FAPs, with its overexpression significantly enhancing adipogenic differentiation [56]. After cells have reached the preadipogenic state, the nuclear hormone receptor PPAR-γ emerges as a master of adipogenesis regulator [57]. PPAR-γ collaborates with transcription factors from the CCAAT/enhancer-binding protein (C/EBP) family to initiate the adipogenic transcriptional program (Figure 4). Wagyu steers, renowned for their extensive marbling, exhibit higher expression of C/EBP family members compared to the less marbled Holstein breed [58]. In mature adipocytes, PPAR-γ continues to be expressed and serves as a marker of adipogenesis, along with lipogenic genes like fatty acid-binding protein 4 (FABP4), perilipin, and fatty acid synthase (FAS). As adipogenesis advances through the cell cycle, mature adipocytes eventually enter a state of growth arrest [59].

### 3.3. Differentiated Mature Cells

Mature cells have limited ability to proliferate and can only be utilized for cultured meat production if they undergo genetic modification or are immortalized [60]. Immortalization involves the loss of cell cycle checkpoint regulation and activation of senescence bypass. In addition to identifying naturally immortalized cell lines, cells can be artificially immortalized by disrupting the P53/P14/RB pathway or by inducing the expression of the catalytic subunit of telomerase (TERT) through the activation of specific viral genes [61]. These methods produce genetically modified cells, which must be labeled appropriately. Immortalized cells may exhibit tumorigenic properties, making it essential to select an appropriate immortalization method and routinely assess the cells to ensure safety. The mature cells that can be used for cultured meat production include adipocytes (which can transdifferentiate into myocytes), myocytes, endothelial cells, and fibroblasts. For example, Believer Meats (formerly Future Meat) has utilized spontaneously immortalized chicken fibroblasts in the production of its cultured meat [62]. Similarly, Upside Foods uses myoblasts derived from fibroblast-like cells and muscle tissue from the skin of mid-stage fertilized chicken eggs, both of which are either spontaneously or artificially immortalized to drive their cultured meat production.

### 3.4. Stem-Cell Genetic Modification

**Figure 5:**
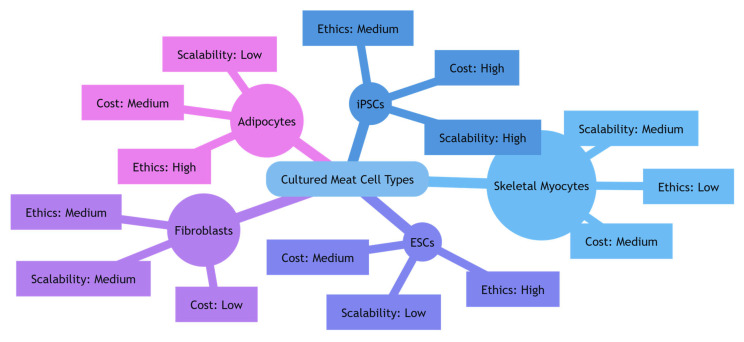
Circular schematic of cell types used in cultured meat production. Each type is linked to three attributes—cost, scalability, and ethics—highlighting key differences in their suitability for large-scale manufacturing.

Genetic modification, whether naturally occurring or induced through genome editing tools, enables the inheritance of traits across successive cell generations. In the context of cultured meat, genetic modification can improve cell expansion or differentiation processes and holds significant potential for various other applications. In contrast, primary cell lines have a limited ability to proliferate [63]. Cell lines derived from adult tissues can become immortalized through genetic marker drift during in vitro culture, as demonstrated by the murine myogenic cell line C2C12 [64]. While genetic drift can occur naturally, it can also be artificially accelerated by applying non-lethal stressors, with ultraviolet irradiation being one of the most commonly used methods [65]. Osmotic stress can induce mutations by enabling the production of immortalized tilapia cell lines [66]. However, spontaneous mutations can produce unpredictable outcomes and require extensive evaluation. For instance, mutations that cause uncontrolled myoblast proliferation may lead to rhabdomyosarcoma formation, which subsequently inhibits differentiation [67]. The selection of cell source species can influence the ability to acquire beneficial mutations, as larger animals have a lower tolerance for selectable mutations. For instance, elephants have multiple copies of the p53 tumor suppressor gene, which enhances the accuracy of DNA synthesis [68]. Moreover, a newly discovered spontaneously immortalized chicken fibroblast line exhibited the capacity to form high-density suspended cell cultures [62]. Although these cells lacked myogenic properties, they were capable of undergoing adipogenesis. Similarly, MuSCs extracted from mackerel have also shown spontaneous immortalization and can differentiate through both myogenesis and adipogenesis [69]. When combined with targeted genome editing [61], cell immortalization presents a highly potential approach for the future of cultured meat, enabling greater cell utility and functionality. To provide a clearer comparison, Figure 5 presents a circular schematic of the main cell types considered for cultured meat production. The diagram highlights differences in cost, scalability, and ethical considerations, offering a visual summary of their relative advantages and limitations.

### 3.5. Critical Comparison of Cell Sources

While genetic modification and immortalization provide promising strategies to improve scalability, the choice of cell type remains central to industrial feasibility and public acceptance. Pluripotent stem cells (ESCs and iPSCs) offer unlimited self-renewal and differentiation capacity, making them attractive for large-scale applications; however, ESCs raise ethical concerns related to embryo use, and iPSCs may face higher costs and technical challenges for standardization. Adult stem cells such as MuSCs and MSCs are more ethically acceptable and perceived by consumers as “closer to natural meat,” but their limited proliferation and differentiation capacity restrict long-term scalability. Immortalized cell lines circumvent these issues by ensuring continuous supply, yet concerns about genetic stability and the potential perception of being “unnatural” could impact consumer acceptance and regulatory approval. Thus, future strategies must balance scalability and functionality with ethical and societal considerations to ensure industrial adoption of cultured meat.

## 4. Factors Affecting the Selection and Performance of Cell Types in Cultured Meat Production

### 4.1. Species

While extensive research has been conducted on culturing common human and mouse cell types, the optimal culture conditions for fish, bovine, and chicken cells remain less explored. Due to the genetic similarities between these animal species, a common basal medium is frequently used, along with comparable growth and proteins factors. In contrast, species-specific biological pathways often need supplementary growth factors, as well as other factors in order to develop these cells and preserve species-specific features. For instance, bovine ESCs rely on the Wnt signaling inhibitor endo (IWR1) to sustain pluripotency and growth, a characteristic that cannot be found in human ESCs [17]. The processes of differentiation of human and mouse cells have biological pathways similar to those of other species, but differences exist in some of the proteins of these pathways. Therefore, understanding the specific pathways and cellular requirements of each species is crucial for optimizing differentiation protocols and identifying the genes and proteins involved in cell development at various stages. Additionally, most commercially available antibodies are designed to target proteins in human or mouse cells and may not effectively cross-react with proteins from other species. Consequently, further research and the development of specialized tools are necessary to accurately monitor and identify the developmental stages of cells from non-human and non-mouse species. In addition, even after species-specific challenges in cell cultivation are addressed, the formulation of the final product and post-processing such as blending muscle and fat components, applying structuring techniques, and ensuring flavor, texture, and safety through conventional food processing remain crucial to delivering consumer-ready cultured meat.

### 4.2. Cultivation Medium

Pluripotent cells require an appropriate environment and a particular set of growth factors to preserve their pluripotency. However, serum-free media formulations for ESC growth are commercially accessible but are more expensive [70]. Similarly, although satellite cells are traditionally cultured with serum, chemically defined and serum-free media have been developed for their cultivation [71]. Eliminating animal serum from the culture processes used for ESC-based cultured meat is believed to improve public acceptance of these products. However, the current regulatory hurdles as alternative reagents must be identified to replace the FBS that is currently used. However, the selection of MSCs as the source of muscle cells offers an advantage by simplifying the development of muscle cell differentiation media. In contrast, producing the desired differentiated cell types from ESCs requires more steps. Therefore, the choice of cell type and species are critical factors that influence media composition, which in turn affects production costs and consumer acceptance of the final product.

### 4.3. Characteristics of the Process and Product

Cells possess epigenetic memory that influences their differentiation potential, depending on the source of the organ biopsy. For example, MSCs derived from bone marrow exhibit a different differentiation capacity compared to those obtained from adipose tissue [72]. It is important to note that it remains uncertain whether cells derived from different breeds will affect the organoleptic properties or production processes of the final product, or if these factors will be influenced by growth conditions and medium composition. In this context, conducting a comparative study on cultured meat produced from cells derived from different bovine breeds, such as Angus, Belgian Blue, Holstein, and Wagyu, would be valuable. Similarly, it is also not clear if the sex of the donor animal would have an impact on the characteristics of the cells and the product. Choosing a cell source for cultured meat involves balancing factors like process complexity, ease of derivation, cost, consumer acceptance, regulatory considerations, and the desired qualities of the final product. To enable comparison across species, Table 2 provides a structured overview of preferred cell sources, culture medium requirements, epigenetic considerations, and industrial feasibility. This summary highlights both commonalities and species-specific differences, offering a clear guide for selecting and optimizing cell types for cultured meat production.

## 5. Overcoming Key Industrial Challenges in Cultured Meat Production

### 5.1. Scale-Up, Automation, and Bioreactors

For cultured meat to become a viable alternative to conventional meat, its production must be scalable and economically sustainable. The scaling approach depends on the intended final product and the number of population doublings that the stem cells can undergo. For example, the scaling requirements for producing minced meat differ significantly from those needed for full-thickness meat products, especially during the advanced stages of tissue or organoid development.

If cell and tissue production are conducted separately, then cell production is likely to follow a similar process. A seed train, consisting of a series of bioreactors with progressively increasing working volumes, is used to sustain cells in a proliferative state. This approach supports the efficient production of the required cell quantities for large-scale manufacturing. It also optimizes resource use by reducing material consumption, feedstock needs, and culture handling. The seed train supports cell expansion from an initial harvest of approximately 10^4^ cells to a final batch of around 10^13^ cells, which is sufficient to produce one ton of cultured muscle meat. Moreover, optimizing the seed train focuses on keeping cells in the exponential growth phase and preventing premature differentiation, which mainly depends on the cell type [85]. Initially, cell cultures are established in standard plates or flasks. As cell numbers expand, they are subsequently transferred to bioreactors, where critical parameters such as pH, temperature, dissolved oxygen, and CO_2_ levels are meticulously controlled to ensure optimal growth conditions.

#### Role of Bioreactors

Bioreactors offer several advantages, including enhanced scalability, precise control over environmental conditions, and the ability to achieve higher cell densities compared to planar culture systems [86]. The key bioreactors used today include stirred-tank bioreactors and rocking or wave bioreactors. Additional bioreactor configurations include packed-bed perfused and fixed-bed reactors, as well as air-lift, hollow-fiber, fluidized-bed, and vertical-wheel bioreactors. Furthermore, new operational modes have been introduced for stirred-tank and rocking bioreactors [87].

The standard industrial approach for culturing mammalian cells relies on stirred-tank bioreactors, which support either suspension cultures or cells adhered to microcarriers suspended in a stirred medium [88]. Most mammalian cells require anchorage for growth; therefore, microcarriers provide a supportive surface for attachment and expansion. Suspension cultures, however, allow higher cell densities and enable more efficient harvesting. For instance, bovine myoblasts, similar to MSCs, can be cultured in suspension using microcarriers. Additionally, advancements have shown some success in cultivating iPSCs as aggregates, comparable to previous achievements with ESCs derived from mice and humans [89]. While some specialized stem cells can form aggregates, maintaining consistent aggregate size is challenging, often leading to variability in cell yields [90]. Currently, large-scale data on culturing cells in aggregates is lacking. Additionally, while cells from the C2C12 myoblast line can form aggregates, they exhibit characteristics of quiescent satellite cells, which limits their capacity for large-scale expansion [91]. The majority of large-scale culturing techniques for anchorage-dependent mammalian cells are currently being advanced in the area of MSC therapy [92].

Each type of bioreactor has its advantages and disadvantages. Stirred-tank bioreactors are commonly used for mammalian cell culture due to their scalability. However, the mechanical agitation required for adequate mixing can subject cells to high shear stress, potentially affecting cell viability and productivity. Moreover, hollow-fiber bioreactors enable cell growth on the outer surface of microfibers or within the hollow spaces between fibers, allowing nutrients to diffuse from the fiber lumen. While this design helps minimize shear stress, it is limited by high operational costs and single-use restrictions [93]. Although hollow-fiber bioreactors enable high cell densities, the attempt to replicate in vivo conditions often results in gradients of waste products, nutrients, pH, and dissolved oxygen, thus limiting scalability. Similarly, in packed or fixed-bed bioreactors, mass transport limitations hinder the uniform distribution of cell quality and viability across the reactor [94]. In contrast, fluidized-bed bioreactors support high-density cell culture through fluidization and medium circulation for mixing by eliminating the need for mechanical agitation. However, these systems have only been scaled to 100 L, and it remains uncertain whether this level of productivity can be achieved in larger vessels.

The primary objective of bioreactor design is to optimize the medium conversion ratio, which represents the proportion of nutrients in the culture medium converted into edible animal tissue, similar to the feed conversion ratio in conventional livestock farming. Recycling techniques can enhance cell density by increasing the number of cells per milliliter of medium while improving medium retention and overall efficiency. Another crucial aspect of bioreactor optimization is scaling up cell production to enhance cost-effectiveness. Tissue formation is equally important, which is essential for producing structured meat products. Moreover, in systems that lack the capability to simultaneously cultivate cells and tissues through self-assembly, a separate in situ bioreactor is required to provide the necessary conditioning for in vivo or preconditioned tissue development, ensuring efficient tissue maturation and integration. The variety of bioreactors designed for tissue formation is expected to be extensive, as each tissue type has distinct condition requirements. To reduce production costs and minimize the risk of microbial contamination, it is essential to automate specific labor-intensive steps within the process.

Furthermore, the primary objective of bioprocess optimization and development is to lower the production costs. In silico modeling of cell behavior is expected to play a crucial role in optimizing large-scale production in the near future. This is particularly important when using primary cells as source material, as substantial efforts are required to move beyond semi-scaled systems and the prevailing trial-and-error approaches currently used in the cell and gene therapy industry [95]. Additionally, the manufacturing process extends beyond cell and tissue production to include cell purification and harvesting, as well as cell banking, storage, and transportation. Other critical considerations include ensuring traceability and standardizing tissue collection from animal donors, maintaining stringent quality control over the produced tissues, and integrating traditional food processing techniques to transform these tissues into meat products. The comparative features of various bioreactor platforms are presented in Table 3, outlining their working scales, advantages, limitations, cost implications, and application relevance. This synthesis provides a structured basis for assessing the technological suitability of different systems and underscores the practical considerations that must be resolved to enable industrial-scale implementation of cultured meat.

### 5.2. Role of Biomaterials

Cellular agriculture depends on biomaterials to support anchorage-dependent cells by providing a scaffold for their expansion and differentiation. These biomaterials not only enable the transport of oxygen and nutrients while enabling the removal of metabolic waste but also prevent the formation of necrotic cores within the tissue. Additionally, achieving an optimal balance between morphology, structure, and chemistry is crucial to ensuring proper cell growth and function. While scaffolds were originally developed for tissue engineering and regenerative medicine, their application in cellular agriculture for food production requires distinct modifications to meet industry-specific demands [102] (Table 2). Importantly, scaffolds are typically degradable. However, for non-degradable scaffolds, it is essential that they are safe for human consumption in both raw and cooked forms. These scaffolds must also meet specific criteria regarding texture, taste, cooking characteristics, and nutritional properties. Additionally, thermal stability is an important consideration to ensure that they maintain their integrity during cooking processes. Scaffolds used in food production must be not only safe and effective but also affordable and scalable for large-scale manufacturing.

#### 5.2.1. Options for Scaffold

Biomaterial scaffolds for cell-based foods are primarily derived from biological sources. These materials are processed into suitable shapes and morphologies while preserving their intrinsic chemical composition. In order to minimize costs, the handling of biologically derived materials should be limited. Moreover, the materials obtained from farmed animals should be avoided, including collagen, because they are not replicating and remain dependent on livestock production. Consequently, polysaccharides like starch (amylopectin/amylose), cellulose, chitosan/chitin, alginates, pullulan, and hyaluronic acid are regarded as more appropriate [103]. Protein-based systems produced through recombinant technology also hold potential, incorporating materials such as gelatin, collagen, fibrin, silk, or keratin. Another promising class of materials is polyesters, including polyhydroxyalkanoates, which can be produced in bacterial and other biological systems [104]. In addition, complex composite matrices sourced from plants and microorganisms, such as decellularized plant leaves, lignins, and fungal mycelia, have been actively studied [105]. Synthetic polymers, including a range of polyesters, are another option for biomaterial scaffolds. These materials are typically biocompatible, safe for human use, and allow for adjustable degradation rates through chemical hydrolysis [106]. Synthetic polymer systems provide benefits like reliable supply and quality, but their cost and the need for surface functionalization can present certain challenges. For bioprinting, applications biomaterials must also meet certain requirements to work effectively as bio-inks.

#### 5.2.2. Evaluation and Methodological Factors

For cellular agriculture, scaffolds require specific properties including texture, digestibility, cooking loss, water binding capacity, and flavor, which are not always the prominent features to select when designing scaffolds for medical applications. All these properties need to be assessed with appropriate methodologies to guarantee the suitability for human consumption in food products. Additionally, nutritional analysis, including the extraction and chromatographic quantification of macronutrients, should be conducted. It is also crucial to assess mechanical properties to evaluate the scaffold’s structure, using methods such as Warner-Bratzler shear force, water retention, and cooking loss (terms commonly used in the meat industry). Furthermore, 3D printing techniques offer the ability to control the morphology of scaffolds, including surface topology, fiber size, porosity, and alignment, ensuring their suitability for the desired applications.

#### 5.2.3. Further Factors to Consider

Cultured meat applications must ensure that the products are both stable and digestible. This can be most effectively evaluated through in vitro testing that simulates gastrointestinal conditions, including pH, mechanical forces, and digestive enzymes. The scaffolds should be evaluated in both their pre- and post-thermal modification (cooked) states to enable a comparative analysis, similar to the assessment methods used for other novel food ingredients [107]. The cost of scaffolding is a critical consideration, as it should represent only a small fraction of the total production cost to ensure consistent product quality, reproducibility, and economic feasibility. Table 4 provides an overview of various polymers that are already produced at an industrial scale.

## 6. Regulation and Safety Aspects of Cultured Meat

In late 2020, the Singapore Food Agency (SFA) became the first regulatory authority to approve a food product derived from cultured animal cells, authorizing the use of cultured chicken cells in food applications. Just Eat’s chicken product, composed of 70% cultured chicken cells and 30% plant protein, was evaluated and considered safe for human consumption at the specified composition levels. Furthermore, the SFA has established a three-step approach to evaluate the safety of cultured meat products. The process begins by assessing the suitability of individual inputs used in the final product and production process, including culture media, cell lines, and reagents. This assessment is supported by toxicological reports for each component to ensure safety and compliance with regulatory standards. Subsequently, it examines the controls and production process to ensure there is no contamination. Lastly, it guarantees that the product complies with food regulations by ensuring that the limits on additives, heavy metals, and allergenic protein levels are consistent with those imposed on traditional meat sources [109].

Additionally, in November 2022, UPSIDE Foods became the first company in the United States to successfully complete a voluntary pre-market consultation for a food product made from cultured animal cells [110]. As part of this process, the Food and Drug Administration (FDA) approved the company’s final product and production process after a comprehensive evaluation. This included assessing potential environmental contaminants, such as microbial and heavy metal contamination; conducting a thorough nutritional analysis; and comparing the product with traditional poultry data. Moreover, all ingredients used in cultured meat production must either be quantified in the final product or evaluated for potential risks to ensure their safety. To promote transparency, UPSIDE Foods’ application, containing non-confidential information about the production and safety of cultured chicken, has been made publicly available [111]. This document states that the nutrient composition of UPSIDE Foods’ cultured chicken has been analyzed and is within expected and safe ranges. The pre-market consultation was concluded after all relevant questions were addressed, with the FDA confirming that it had no further questions regarding the company’s safety assessment.

Certain cell lines used in the production of UPSIDE Foods’ cultivated chicken are immortalized through genetic modification by continuously expressing the chicken TERT gene [106]. According to UPSIDE Foods, the targeted genetic modification of poultry cells via cisgenesis presents a safe and viable alternative to conventional poultry meat [112]. Unlike traditional genetic modification, the cisgenic approach involves extracting and reintroducing genes already present within the genome while modifying their expression. This method specifically activates an endogenous cellular pathway that naturally exists in normal tissues. Furthermore, the company highlighted that, unlike plants, animal cells commonly consumed as food do not typically produce or contain toxins. Additionally, UPSIDE Foods emphasized that the risks associated with off-target effects or pleiotropy in animal cells are considered minimal to negligible. Building on this advancement, in March 2023, GOOD Meat’s chicken cell-based product became the second cultured animal cell product to undergo a pre-market consultation [110].

The voluntary pre-market consultation process is distinct from the FDA’s regulatory framework for gene editing in whole animals. Under this framework, any intentional genomic modification, including cisgenic alterations, is classified as an unapproved drug. As a result, such modifications require approval as a new animal drug before being used in food applications. This regulatory requirement can substantially extend the approval timeline and increase the costs associated with the commercialization of meat derived from genetically engineered animals [113].

Before cultured meat can be commercially sold, the cell manufacturing facility must obtain a grant of inspection from the U.S. Department of Agriculture (USDA) Food Safety and Inspection Service (FSIS) for its harvesting and post-harvest processing operations. Additionally, the final product must display the USDA mark of inspection to ensure compliance with regulatory standards. To establish a structured regulatory framework, a formal agreement was reached in 2019 between the FDA and USDA-FSIS to implement joint oversight of cultured cell-based food products derived from poultry, livestock, and catfish [114]. Unlike conventional meat-processing plants and abattoirs, which require on-site USDA inspectors, cultured meat facilities fall under the inspection authority of the FDA. However, the USDA maintains regulatory control over the processing and labeling of cultured meat products, in line with its responsibilities under the Federal Meat Inspection Act and the Poultry Products Inspection Act [115]. Furthermore, under the current U.S. regulatory framework, the FDA has exclusive jurisdiction over cultured cells derived from game meat, seafood (excluding catfish), and food products intended for animal consumption. As a result of this multi-agency oversight, in June 2023, UPSIDE Foods successfully completed the U.S. pre-market regulatory review process for cultured meat by securing a USDA grant of inspection for its cultivated chicken, marking a significant milestone in the commercialization of cell-based food products.

At present, no cell-based food products are available on the European Union (EU) market. Such products fall under the Novel Foods Regulation (Regulation (EU) No. 2015/2283), which defines novel foods as those lacking a significant history of consumption in the EU before 15 May 1997. This regulation clearly includes foods derived from the cultivation of cells or tissues originating from plants, animals, fungi, microorganisms, or algae. Moreover, if genetic engineering is involved in the production process, the products must also comply with the regulations governing genetically modified food and feed (Regulation (EC) No 1829/2003). Before entering the market, cell-based food products must undergo a mandatory pre-market authorization process. This involves submitting an application and undergoing a rigorous safety evaluation conducted by the European Food Safety Authority (EFSA). The EFSA assessment considers multiple factors, including the composition and nutritional properties of the novel food, as well as potential toxicological and allergenic risks. Additionally, the evaluation inspects the intended use and expected consumption levels, production methods, and the additives and ingredients used in the bioreactor. Despite ongoing regulatory developments, as of 1 March 2023, Singapore remains the only country where a cell-based food product has received market approval. Meanwhile, on a global scale, the World Health Organization (WHO) continues to provide an overview of both general and specific regulatory frameworks related to cell-based food products [116]. To provide a clearer overview, Table 5 presents a comparative analysis of regulatory frameworks governing cultured meat across major regions, highlighting similarities and differences in approval processes, labeling requirements, and safety evaluations. This summary enables a deeper understanding of how regulatory diversity may influence product development, innovation, and global trade. The table further highlights the need for consistent international standards to ensure market accessibility and consumer trust.

Despite progress in individual countries, regulatory frameworks for cultured meat remain fragmented, with variations in approval processes, definitions, and labeling requirements posing barriers to global commercialization. Greater international collaboration, including the development of harmonized safety standards and guidelines, offers an opportunity to streamline approval pathways and build consumer trust, thereby facilitating broader market adoption.

## 7. Acceptance by Consumers

Cultured meat presents several social considerations and challenges, including the necessity for appropriate regulatory oversight, potential shifts in power dynamics within the food industry, and the economic impact on communities reliant on traditional animal agriculture [124]. A key concern is whether consumers will accept cultured meat, as their willingness to adopt this technology will significantly influence its initial market success. Furthermore, consumer acceptance will play a crucial role in determining the long-term societal benefits that cultured meat may offer.

Survey data on consumer acceptance of cultured meat vary significantly based on multiple factors, including the phrasing of survey questions and the nationality of respondents [125]. The wording of questions and the composition of the sample population can greatly influence survey outcomes. A crucial determinant of survey results is the level of information provided to respondents. Notably, surveys specifically designed to evaluate perceptions of cultured meat and incorporating detailed, positive information about the technology tend to yield more favorable responses [125]. In contrast, negative perceptions are often associated with shorter surveys that offer minimal context about cultured meat, typically conducted as part of broader omnibus surveys [126]. This pattern aligns with existing evidence demonstrating that exposure to positive or negative information about cultured meat directly shapes consumer attitudes. Furthermore, experimental studies have identified various strategies to enhance consumer acceptance. Research suggests that presenting cultured meat as a technological innovation is less effective than emphasizing its societal benefits or its similarity to conventional meat [125]. Additionally, simpler descriptions resonate more with consumers compared to complex, technical explanations. The choice of terminology also plays a role, as phrases like “clean meat,” which highlight the advantages over traditional meat, tend to be more interesting than terms such as “lab-grown meat,” which may evoke perceptions of artificiality [127]. Beyond messaging, consumer acceptance is also influenced by practical considerations. People are more inclined to adopt cultured meat when it is offered at a competitive price and perceived as socially accepted by others. Importantly, these factors often determine consumers’ initial willingness to try cultured meat, whereas sustained adoption is more likely to depend on repeated positive experiences, perceived safety, and long-term affordability. These findings highlight the importance of strategic communication and market positioning in the development of consumer confidence and mainstream adoption of cultured meat [128].

Research indicates that familiarity with cultured meat technology is a key factor influencing consumer acceptance, whereas individuals with food neophobia are more likely to reject it [129]. Survey data reveal that a significant proportion of Americans (57.3%) are “completely unfamiliar” with cultured meat [129], highlighting a potential barrier to widespread adoption. Furthermore, focus group studies suggest that initial negative perceptions often become less pronounced once participants have had more time to consider the concept [130]. Although longitudinal data on this subject remain limited, it is expected that positive perceptions and consumer willingness to adopt cultured meat will increase over time. Since attitudes are shaped by both supportive and critical information, the nature of the information consumers encounter may play a crucial role in shaping their acceptance [131]. Notably, media coverage of cultured meat has so far been predominantly positive, which may contribute to greater consumer openness and interest in the technology.

Studies consistently demonstrate that younger individuals are more likely to accept cultured meat compared to older generations. Similarly, acceptance tends to be higher among omnivores than vegetarians and among men rather than women [128,132,133]. The observed gender difference may be attributed to women’s generally more cautious approach to food, while the greater openness among younger individuals is likely due to their increased willingness to embrace new experiences [127]. Cross-cultural research further shows that consumer acceptance differs by region: for example, Asian consumers often emphasize food safety and technological innovation, while European consumers are more concerned with naturalness, labeling, and ethical issues. These cultural variations suggest that marketing strategies should be tailored to local values and norms to achieve wider adoption [134,135]. One of the key advantages of cultured meat is its ability to address the primary ethical and environmental concerns that drive people toward vegetarianism [134]. However, despite these benefits, vegetarians often exhibit an emotional aversion to meat, which can overshadow the logical reasons for avoiding it. Thus, while vegetarians are sometimes discussed as a potential consumer base, their generally lower acceptance highlights the need for more nuanced interpretation rather than broad generalizations [136]. Nevertheless, this does not pose a significant challenge for producers or supporters, as vegetarians represent a relatively small segment of the market and do not substantially contribute to the demand for conventional meat. For cultured meat to effectively reduce reliance on traditional meat, it must not be perceived solely as a product intended for vegetarians. Such a perception could limit its appeal to non-vegetarians, thereby lessening its potential to replace conventional meat and mitigate the ethical, environmental, and sustainability issues associated with animal-based meat production.

A fundamental limitation of research on consumer acceptance of cultured meat is its inherently predictive nature. Due to the fact that cultured meat products are not yet commercially available, researchers have been unable to analyze actual consumer behavior or determine which product attributes may influence consumer preference. However, existing studies indicate that attitudes toward cultured meat align with demographic trends observed in the acceptance of genetically modified foods [137]. This parallel suggests that public perceptions of both technologies may be shaped by similar concerns, as some individuals argue that the conceptual similarities between them lead to comparable attitudes and apprehensions [138]. Future work should therefore focus not only on willingness-to-try surveys but also on behavioral experiments, longitudinal tracking, and cross-cultural comparisons that can better distinguish between short-term curiosity and long-term integration of cultured meat into diets.

Beyond consumer perception, broader socioeconomic, ethical, and global disparity considerations must also be acknowledged. Large-scale adoption of cultured meat could disrupt traditional livestock industries, affecting farmers, rural economies, and employment patterns, particularly in low-income regions where animal husbandry plays a central role in livelihoods. Ethical debates extend beyond animal welfare, encompassing questions of corporate concentration, intellectual property control, and whether food production should be dominated by a few high-tech companies. Moreover, global disparities may arise, as high production costs and advanced infrastructure requirements could restrict cultured meat to wealthier nations, limiting its accessibility in regions most affected by food insecurity. Addressing these issues requires inclusive policies, equitable technology transfer, and international cooperation to ensure cultured meat contributes to sustainable food systems without reinforcing existing inequalities.

## 8. Conclusions and Future Perspectives

Cultured meat represents a paradigm-shifting innovation at the intersection of food technology, ethics, and sustainability. Considerable progress has been achieved in demonstrating its potential to reduce environmental burdens, lessen dependence on conventional livestock production, and contribute to global food security; however, significant challenges remain unresolved. From a scientific perspective, critical bottlenecks include the establishment of stable and scalable cell lines, the development of cost-effective and serum-free culture media, advancements in bioreactor design and efficiency, and the identification of scaffolding materials that are both biocompatible and economically viable. On the regulatory side, fragmented approval frameworks, divergent approaches to GMO classification, and lengthy authorization processes continue to create uncertainty, thereby limiting international market expansion. Recent technoeconomic analyses indicate that the cost of cultured meat ranges from approximately US$16 per kg under large-scale optimized conditions to substantially higher levels at current pilot-scale production, primarily due to the expense of growth media. Notable progress has nevertheless been made, with regulatory approvals already granted in Singapore, the United States, and Israel, including the authorization of the first cultivated beef steak in 2024. While unstructured products are currently the closest to commercialization, structured cuts such as steak remain technically feasible but constrained by issues of scale and cost. Production timelines generally range from two to eight weeks, depending on the complexity and type of product.

**Figure 6:**
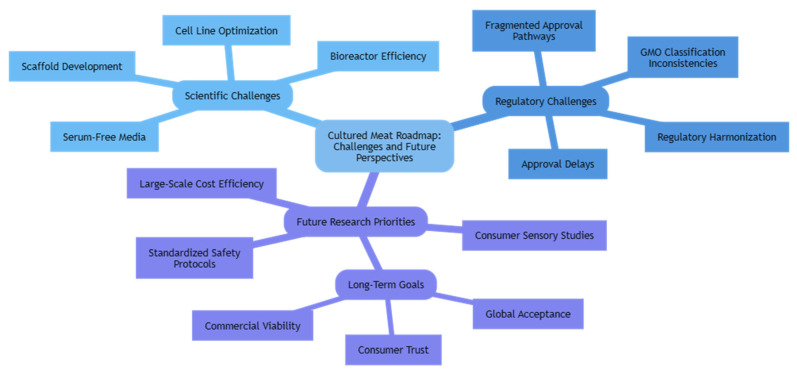
Cultured meat road map challenges and future perspectives.

Future research must adopt an interdisciplinary perspective, integrating advances from cell biology, bioprocess engineering, materials science, consumer science, and regulatory studies. Particular attention should be directed toward the standardization of safety assessment protocols, the economic optimization of large-scale production, the refinement of sensory and consumer acceptance studies, and the pursuit of harmonized regulatory pathways across jurisdictions. Addressing these challenges will not only enable the commercial scalability of cultured meat but also substitute consumer confidence and strengthen its role as a credible contributor to sustainable protein transitions. To provide a visual overview of these challenges and research directions, a roadmap summarizing the scientific, regulatory, and societal priorities for advancing cultured meat is presented in Figure 6.

## Figures and Tables

**Table 1:** Sources of cells for cultured meat [22].

Category of Cell		Position	Differentiation	Proliferation	Advantages	Disadvantages
Pluripotent stem cells (PSCs)	Embryonic stem cells (ESCs)	Cell cluster inside a blastocyst	All three germ-layers	Unlimited	Consistency and reproducibility; formation of cell banks to avoid several biopsies	Genetic stability must be verified; derivation is difficult; intricate differentiation protocols
	Induced PSCs	Somatic cells reprogrammed	All three germ-layers	Unlimited	Compared to embryonic stem cells easier derivation	Intricate differentiation protocols; genetic modification necessary; potential phenotypic changes; epigenetic memory
Adult stem cells (ASCs)	Mesenchymal stem cells (MSCs)	Adipose tissue or bone marrow	Primarily focused on collagen producing cells (fibroblasts), skeletal muscle, and adipocytes	Finite	Easy differentiation into adipocytes	Requires several genetic modifications or biopsies; restricted proliferation
	Fibro-adipogenic precursor cells	Muscle tissue	Osteogenic cells myofibroblasts, chondrocytes, adipocytes	Finite	Easy differentiation into fibroblasts and adipocytes	
	Muscle satellite cells	Muscle tissue	Myoblasts (which eventually develop into myocytes, myotubes, and myofibers)	Finite	The simple differentiation into the primary component of cultured meat, muscle cells	
Mature cells	Adipocytes	Fat tissue	Already distinguished	Finite or none, until immortalized	No specific requirements for differentiation; just basic cultivation	Immortalization or genetic modification may be necessary; concerns regarding regulation and consumer acceptance; immortalization could alter cell properties and decrease differentiation potential
	Myocytes	Muscle tissue	Myofibers, myotubes	Finite or none, until immortalized	No specific requirements for differentiation; just basic cultivation	
	Fibroblasts	Skin, any connective tissue	Fibrocytes, myofibroblasts, adipocytes, chondrocytes, and osteocytes	Finite		
	Endothelial cells	Muscle, skin, or any other blood tissue	Self-assemble into vascular tissues; already differentiated	Finite		
	Smooth muscle cells	Artery tissue	Support blood vessel self-assembly; already differentiated	Finite		

Abbreviations: PSCs, pluripotent stem cells; ESCs, embryonic stem cells; ASCs, adult stem cells; MSCs, mesenchymal stem cells.

**Table 2:** Comparative overview of species-specific cell sources, culture requirements, and feasibility for cultured meat production.

Species	Preferred Cell Sources	Culture Medium Requirements	Epigenetic Considerations	Industrial Feasibility	References
Bovine	Muscle satellite cells (SCs), adipose-derived mesenchymal stem cells (MSCs), immortalized bovine satellite cells (iBSCs).	Serum-free, chemically defined media; ligands for IGF1R, TFRC, LPAR1; engineered iBSCs with TERT/CDK4 for extended proliferation.	Transcriptomic heterogeneity (quiescent, active, committed states); limited locus-specific data.	High feasibility: iBSCs > 120 doublings; serum-free differentiation supports 3D constructs; engineered muscle–fat tissues demonstrate improved structure and cost-saving strategies.	[73,74,75,76,77,78,79]
Porcine	Muscle stem cells (SCs) enriched via cluster of differentiation 29/56 (CD29/CD56) sorting; adipose-derived mesenchymal stem cells (ADSCs); Wharton’s jelly mesenchymal stem cells (WJ-MSCs).	Skeletal muscle growth medium-2 (SkGM-2) supplemented with epidermal growth factor (EGF), dexamethasone, and p38 mitogen-activated protein kinase inhibitor (SB203580); extracellular matrix (ECM) modulation with connective tissue growth factor (CTGF) promotes myogenesis.	Adipose-derived stem cells (ADSCs) show higher proliferation and adipogenesis; Wharton’s jelly mesenchymal stem cells (WJ-MSCs) exhibit greater osteogenic potential; limited epigenetic mapping available.	Magnetic-activated cell sorting (MACS) enrichment raises CD56+/CD29+ muscle stem cells (SCs) to ~91%; adipose-derived stem cells (ADSCs) expand efficiently and support scale-up; extracellular matrix/connective tissue growth factor (ECM/CTGF) methods are promising but require large-scale validation.	[80,81,82]
Chicken	Muscle satellite cells (SCs), mesenchymal stem/progenitor cells (MSCs); induced pluripotent stem cells (iPSCs) as alternatives.	Serum-free, species-adapted media with optimized growth factors and supplements needed; no validated chicken-specific formulations yet.	No chicken-specific DNA methylation or histone modification data; only general stem/progenitor control discussed.	Similar challenges to mammals: limited SC expansion, lack of stable cell lines, and costly media; no large-scale demonstrations available.	[83,84]
Fish	Reviews highlight fish as potential cultured meat (CM) targets, but specific protocols and quantitative culture data are scarce.	No validated fish-specific serum-free formulations or standardized medium recipes reported.	No fish-specific DNA methylation or histone modification studies available.	Similar challenges to mammals: limited expansion of adult stem cells (ASCs), absence of reliable cell lines, and costly media; no large-scale demonstrations of fish muscle or fat production exist.	[83,84]

**Table 3:** Comparative overview of bioreactor platforms for cultured meat production, highlighting working scales, advantages, limitations, cost, and applications.

Bioreactor Type	Typical Working Scales	Key Advantages	Major Limitations	Quantitative Examples	References
Stirred tank reactor (STR)	Laboratory to industrial; single-use and stainless steel up to tens–hundreds of thousands L in conceptual designs	Well-established industrial platform; good process control, oxygen transfer and mixing; compatible with microcarriers and perfusion	Shear sensitivity for adherent cells, oxygen transfer limits at high cell density, high facility CAPEX for sterile food-grade operations	Facility scenarios modeled with ~42,000 L and ~211,000 L STRs; COGS scenarios show ~35/kg and 35/kg and 25/kg, respectively, in technoeconomic models	[96]
Perfusion (cell retention in stirred systems)	Lab to pilot; limited high-density perfusion volumes industrially	Sustains high viable cell concentrations by continuous nutrient supply and waste removal	Complex cell retention hardware, scale limits from perfusion rates and mass transfer, increased media consumption	Perfusion operation and cell density tradeoffs are central scale-up constraints noted in technoeconomic and scale-up analyses	[96,97]
Microcarrier-based cultures	Lab to pilot; scalable in STRs (single use vessels ~1–10,000 L)	Converts adherent cells to high-surface-area 3D growth in agitated vessels; enables bead-to-bead transfer for intensification	Microcarrier cost, downstream separation, shear and bead collisions, harvesting efficiency	Intensified microcarrier process achieved ~114× fold expansion at liter scale using bead-to-bead and stepped additions	[98]
Fixed-bed/packed-bed	Lab to small pilot	High surface area per unit volume for adherent cells and scaffold integration; low shear	Channeling, poor mixing, difficult uniform oxygenation at larger scales, limited sampling	Application discussed conceptually for scaffolded tissues; quantitative scale/density not reported in corpus (insufficient evidence)	[99]
Fluidized bed	Lab to small pilot	Improved mass transfer around particles/carriers, potentially uniform exposure	Particle abrasion, carrier retention hardware, shear on cells, scale-up complexity	Mentioned as candidate in reviews but no quantitative CM data available in supplied corpus (insufficient evidence)	[99]
Hollow fiber bioreactor (HFB)	Lab to prototype; small pilot for tissue constructs	Very high local mass transfer via semi-permeable fibers; enables perfusable, aligned tissues and centimeter-scale constructs	Complex geometry, limited homogeneous large-volume manufacture, fiber fouling, difficult scale-out	Centimeter-scale perfusable muscle constructs produced with HFB and active perfusion, improving maturation and texture in lab studies	[100]
Airlift reactor	Conceptual pilot to large industrial in models	Low shear mixing, good gas handling, lower energy input; modelled as viable large-scale alternative	Lower power for mixing may limit mass transfer at very high cell densities; less industrial experience for adherent mammalian cells	A scenario with ~262,000 L airlift reactor gave projected COGS ~$17/kg in a technoeconomic model	[96]
Macrofluidic single-use systems	Laboratory and R&D prototyping; potential scale-out	Cheap, rapidly prototyped, food-grade thermoplastic assemblies reduce equipment cost and contamination risk	Limited to small/medium volumes per unit; needs scale-out with many parallel units	Laser-welded polyethylene macrofluidic single-use bioreactors demonstrated scaffold cultivation and reduced prototyping cost	[101]

**Table 4:** Polymer alternatives for scaffolds in cellular agriculture from non-animal sources [108].

Class of Biopolymers	Origin and Characteristics	Specific Category
Polysaccharides	Bacteria, plants	Derivatives of cellulose and cellulose (MC, HMPC, CMC)
	Plants	Amylopectin, amylose (starch)
	Yeast, fungi, insects, crustaceans	Chitosan/chitin
	Heterologous-expression	Methacrylate derivatives, hyaluronic acid
	Plants	Agarose
	Plants	Alginate
Proteins	Heterologous expression	Zein, collagen/gelatin, Methacrylate derivatives
	Heterologous expression	Keratin
	Heterologous expression	Elastin
	Heterologous expression	Laminin
Synthetics	Chemical synthesis	Polyglycol acids/polylactic
	Chemical synthesis	Polyethylene glycol
	Chemical synthesis	Polycaprolactone
	Chemical synthesis	Polyvinyl–alcohol
Polyesters	Heterologous expression	Polyhydroxyalkanoates, including homopolymers and copolymers
Complex natural composites	Plant	Lignin
	Fungi	Mycelia
	Plants	Soy hydrolysates
	Plants	Decellularized tissues

Abbreviations: MC, methylcellulose; HPMC, hydroxypropyl methylcellulose; CMC, carboxymethyl cellulose.

**Table 5:** Comparative assessment of cultured meat regulation in key regions.

Country	Approval Status	Regulatory Pathway	Key Agencies	Major Regulatory Steps	Labeling Requirements	GMO Classification	Approval Timeline	Critical Challenges	References
Singapore	Market Access Granted	Novel Food Assessment	Singapore Food Agency (SFA)	1. Pre-market consultation; 2. Safety dossier submission; 3. Technical review; 4. Risk assessment; 5. Market authorization.	“Cell-cultured” or “cultured” labeling required	Case-by-case assessment for GMO components	12–18 months (estimated)	Limited precedent, evolving guidelines	[117,118,119]
United States	Limited Approvals	Dual Agency Model (FDA/USDA)	FDA (cell culture safety); USDA-FSIS (inspection)	1. FDA pre-market consultation; 2. Generally Recognized as Safe (GRAS) determination; 3. USDA facility inspection; 4. Hazard Analysis Critical Control Points (HACCP) plan; 5. Label approval.	“Cell-cultured” required in product name	Follows existing GMO labeling rules (voluntary disclosure)	18–36 months	Agency coordination, unclear jurisdiction boundaries	[118]
European Union	No Approvals	Novel Food Regulation	EFSA (scientific assessment); European Commission Member States	1. Novel food application; 2. EFSA scientific opinion; 3. Risk management decision; 4. Member state consultation; 5. Commission authorization.	Must comply with novel food labeling; clear indication of production method	Subject to GMO regulations if applicable; mandatory labeling if GMO components	24–48 months	Complex multi-stakeholder process, precautionary approach	[119,120,121]
Israel	Regulatory Progress	National Food Safety Framework	Ministry of Health; Israeli Food Service	1. Pre-market notification; 2. Safety assessment; 3. Production facility approval; 4. Product authorization.	Hebrew labeling requirements; clear production method indication	Follows national GMO framework	12–24 months (estimated)	Limited regulatory precedent	[122]
Japan	Under Development	Food Safety Framework (evolving)	Ministry of Health, Labour and Welfare (MHLW); Food Safety Commission	1. Safety assessment consultation; 2. Technical review; 3. Risk evaluation; 4. Authorization process.	Japanese labeling standards; production method disclosure	Subject to existing GMO regulations	TBD	Regulatory framework still developing	[123]
China	Policy Development	National Food Safety Standards	National Health Commission; China Food and Drug Administration	1. New food ingredient application; 2. Safety evaluation; 3. Technical review; 4. Administrative approval.	Chinese labeling requirements; production method indication	Strict GMO labeling requirements	TBD	Regulatory framework in early stages	[119]

## Data Availability

No new data were created or analyzed in this study. Data sharing is not applicable to this article.

## Abbreviations

The following abbreviations are used in this manuscript:

ADSCs	Adipose-derived stem cells
ASCs	Adult stem cells
C/EBP	CCAAT/enhancer-binding protein
CD	Cluster of differentiation
CMC	Carboxymethyl cellulose
ECM	Extracellular matrix
EFSA	European Food Safety Authority
ESCs	Embryonic stem cells
FABP	Fatty acid-binding protein
FACS	Fluorescence-activated cell sorting
FAPs	Fibro-adipogenic progenitor cells
FAS	Fatty acid synthase
FBS	Fetal bovine serum
FDA	Food and Drug Administration
FSIS	Food Safety and Inspection Service
GAPDH	Glyceraldehyde-3-phosphate dehydrogenase
GFs	Growth factors
HESCs	Human embryonic stem cells
HSCs	Hematopoietic stem cells
HPMC	Hydroxypropyl methylcellulose
IGF1	Insulin-like growth factor 1
iPSCs	Induced pluripotent stem cells
MSCs	Mesenchymal stem cells
MuSCs	Muscle satellite cells
MYF5	Myogenic factor 5
MYOD	Myogenic differentiation 1
MYOG	Myogenin
OPTi-OX	Optimized transcription and expression system (proprietary technology by Meatable)
PAX7	Paired box 7
PPAR-γ	Peroxisome proliferator-activated receptor gamma
PSCs	Pluripotent stem cells
PUFAs	Polyunsaturated fatty acids
SFA	Saturated fatty acids
TERT	Telomerase reverse transcriptase
Tn	Troponin
Tm	Tropomyosin
WHO	World Health Organization
Zfp423	Zinc finger protein 423
